# Anticancer Activity of *Amauroderma rude*

**DOI:** 10.1371/journal.pone.0066504

**Published:** 2013-06-20

**Authors:** Chunwei Jiao, Yi-Zhen Xie, Xiangling Yang, Haoran Li, Xiang-Min Li, Hong-Hui Pan, Mian-Hua Cai, Hua-Mei Zhong, Burton B. Yang

**Affiliations:** 1 Guangdong Institute of Microbiology, Guangzhou, China; 2 Sunnybrook Research Institute, Sunnybrook Health Sciences Centre, Toronto, Canada; 3 Department of Laboratory Medicine and Pathobiology, University of Toronto, Toronto, Canada; University of North Carolina School of Medicine, United States of America

## Abstract

More and more medicinal mushrooms have been widely used as a miraculous herb for health promotion, especially by cancer patients. Here we report screening thirteen mushrooms for anti-cancer cell activities in eleven different cell lines. Of the herbal products tested, we found that the extract of *Amauroderma rude* exerted the highest activity in killing most of these cancer cell lines. *Amauroderma rude* is a fungus belonging to the *Ganodermataceae* family. The *Amauroderma* genus contains approximately 30 species widespread throughout the tropical areas. Since the biological function of *Amauroderma rude* is unknown, we examined its anti-cancer effect on breast carcinoma cell lines. We compared the anti-cancer activity of *Amauroderma rude* and *Ganoderma lucidum*, the most well-known medicinal mushrooms with anti-cancer activity and found that *Amauroderma rude* had significantly higher activity in killing cancer cells than *Ganoderma lucidum*. We then examined the effect of *Amauroderma rude* on breast cancer cells and found that at low concentrations, *Amauroderma rude* could inhibit cancer cell survival and induce apoptosis. Treated cancer cells also formed fewer and smaller colonies than the untreated cells. When nude mice bearing tumors were injected with *Amauroderma rude* extract, the tumors grew at a slower rate than the control. Examination of these tumors revealed extensive cell death, decreased proliferation rate as stained by Ki67, and increased apoptosis as stained by TUNEL. Suppression of c-myc expression appeared to be associated with these effects. Taken together, *Amauroderma rude* represented a powerful medicinal mushroom with anti-cancer activities.

## Introduction

Natural products have attracted extensive attention not only in health promotion and disease treatment but also in drug discovery and development. The natural product-based drug discovery and development are still one of the major routes leading to the development of therapeutics for various diseases including cancer. In the area of cancer and infectious diseases, more than half of the drugs have natural origins. Natural products have some forms of biological activity because they contain low concentrations of the active ingredients. Thus, many drugs are obtained directly from natural sources, especially in cases where there are bioactive compounds with complex structures, making the synthesis difficult. On the other hand, some drugs are developed based on the lead compounds of the natural source or new analogues are designed to serve the clinical purpose. Usually, natural products are obtained from plant kingdom, animals, and microbial world. Microorganisms have been invaluable for drug discovery and development of lead compound-based drugs because certain bioactive molecules can only be obtained from particular organisms. Medicinal mushrooms are a large group of organisms that are extensively used as antiviral, antimicrobial, anti-inflammatory, antihyperglycemic, and anticancer compounds.

*Ganoderma lucidum*, a traditional Chinese medicinal fungus, is the most famous medicinal mushroom. For over 2000 years, it has been used in traditional Chinese medicine as a medicinal mushroom. This medicinal mushroom has been regarded as folk medicine used for the prevention and treatment of various human diseases, including chronic bronchitis, hepatitis, hypertension, hypercholesterolemia, immunological disorders, and cancers [1]. It has been known that the major bioactive ingredients in *Ganoderma lucidum* are polysaccharides, ganoderic acid (triterpene), and adenosine. The polysaccharides from *Ganoderma lucidum* possess biological activity and are of therapeutic application [2]–[6], while ganoderic acid possesses anti-tumour and anti-HIV-1 activities [7], [8], in addition to other biological activities including facilitating histamine release [9], cytokine production [10], and immunomodulatory activity [11]. *Ganoderma lucidum* is the most well studied member of the *Ganodermataceae* family. This family contains 11 genus including Amauroderma. As a genus of *Ganodermataceae*, Amauroderma is widespread in tropical areas and contains about 30 species [12] **.** Among them, *Amauroderma rude* is a newly described fungus in 2007 (http://australianfungi.blogspot.ca/2007/04/7-amauroderma-rude.html). This mushroom is brown with concentric zones of varying shades on the cap. Named ‘Xuezhi’ in China, translated to bloody mushroom, this mushroom can be cultivated in the same farm that produces *Ganoderma lucidum* for our studies [13], [14]. When screening bioactive medicinal mushrooms for anti-cancer activity, we unexpectedly found that *Amauroderma rude* possessed the highest activity in inducing cancer cell death. We thus designed a series of experiments to characterize the anti-cancer effects of this mushroom.

## Results and Discussion

### *Amauroderma rude* Extract Exerts the Highest Activity in Inducing Cancer Cell Death

**Figure 1:**
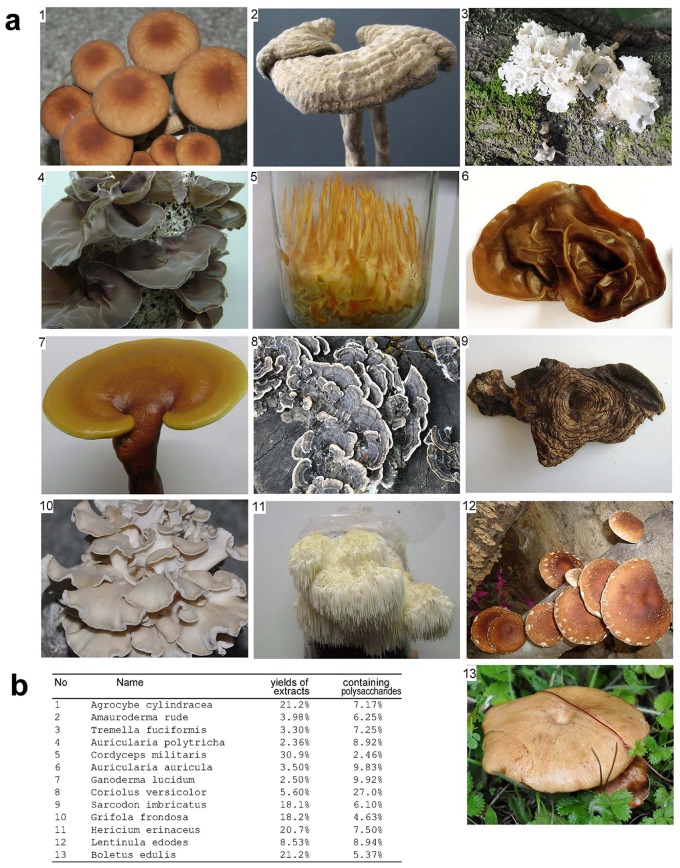
Preparation of thirteen types of mushrooms. (a) Photograph of thirteen types of mushrooms used in the study. (b) The thirteen types of mushrooms were dried and subject to hot water extract. The concentrations of polysaccharides were also measured.

We have previously reported that *Ganoderma lucidum* possessed anti-cancer cell activity [13], [15]. Extract from *Ganoderma lucidum* inhibit cancer cell adhesion by reducing integrin expression [16] and can inhibit tumor cell proliferation [17]. Recent study also reported an anti-tumor effect of *Ganoderma lucidum* extract in breast cancer models by inhibiting protein synthesis and tumor growth [18]. In this study, we screened the anti-cancer cell activity of 13 types of popular mushrooms (Fig. 1a). The fruit bodies of all mushrooms were dried and subject to isolation of biologically active components by hot water extraction. It was found that the extract rates of different types of mushrooms were very different, reaching more than 10-fold difference (Fig. 1b). It has been reported that polysaccharides are the major components in the water extracts that possess anti-cancer-activity [19]–[21]. We measured the concentrations of polysaccharides in all extracts and found that the levels of polysaccharides varied greatly.

**Figure 2:**
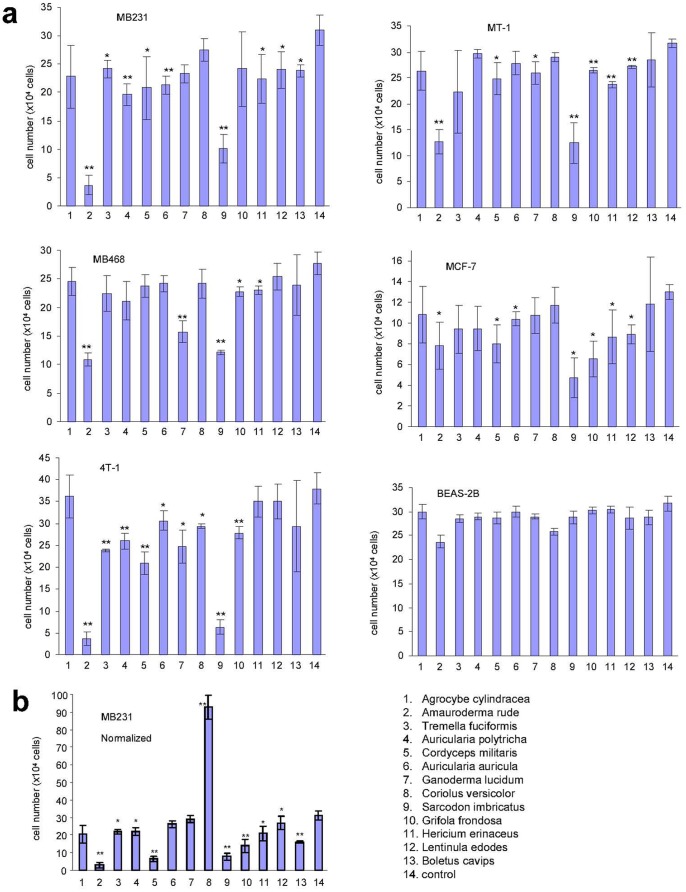
Effects of the thirteen mushroom extracts on breast cancer cell activity. (a) Cells of MDA-MB231, MDA-MB468, MT-1, MCF-7, 4T1, and BEAS-2B were inoculated in 24-well tissue culture plates at a density of the 8×10^4^ cells/well. Four hours after cell inoculation, extracts from the thirteen different mushrooms were added to each well at a concentration of 400 µg/ml and incubated at 37°C in a tissue culture incubator for 48 hours. Cell survival was determined by trypan blue staining and cell counting. n = 3, *p<0.05; **p<0.01. (b) The number of cells was normalized to the same concentration of polysaccharides listed in Figure 1b using MB231 cells to examine the pattern of effects of total extract and polysaccharides.

Since the total extracts are normally the components used especially for the woody medicinal mushrooms, we screened the anti-cancer effect of the extracts. We examined the effect of these extracts on the most invasive and metastatic breast cancer cell lines including three human breast carcinoma cell lines, MDA-MB231 (purchased from ATCC), MDA-MB468 (from ATCC), and MT-1, originally established by Engebraaten and Fodstad [22], and one mouse breast cancer cell line 4T1, originally established by Aslakson and Miller [23]. Amongst these cell lines, MDA-MB468 was the least invasive. A benign human breast cell line MCF-7 (from ATCC) and a normal lung cell line BEAS-2B (from ATCC) were used as controls. A buffer vehicle served as a negative control for each assay. We found that *Amauroderma rude* was the most effective agent in inducing cancer cell death (Fig. 2a). Interestingly, the level of Coriolus versicolor was extremely high, but we did not detect a comparable effect of this mushroom on inducing cancer cell death. We thus normalized the number of cells to the same concentration of polysaccharides and found that the patterns of effects played by the extract and polysaccharides varied greatly (Fig. 2b). This deviation suggests that the polysaccharides are not the major component in the medicinal mushrooms possessing anti-cancer cell activity. The non-polysaccharide-fraction may play essential role in inducing cancer cell death. Indeed, we have previously reported that a small molecule ergosterol peroxide isolated from *Ganoderma lucidum* has strong anti-cancer activities [13]. Fractionation and purification are needed to isolate the essential components possessing the anti-cancer cell activities.

**Figure 3:**
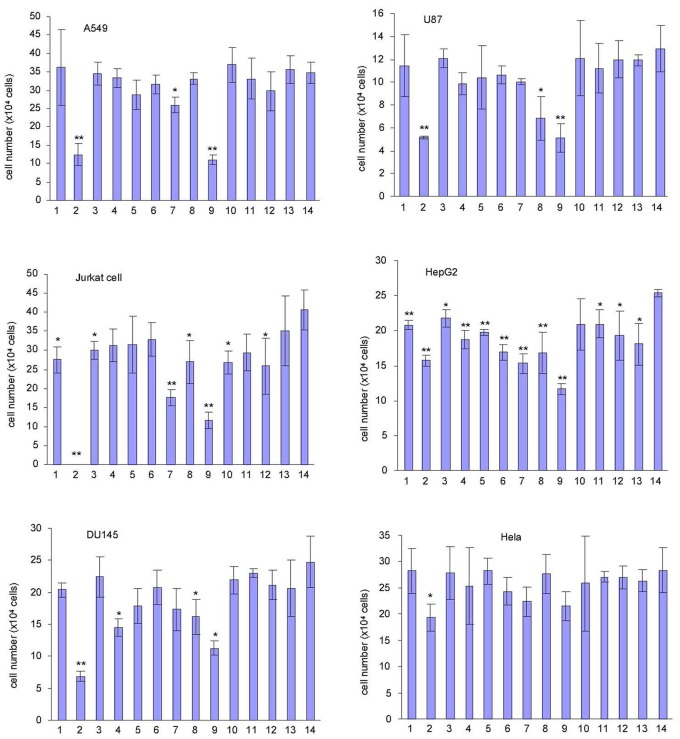
Effects of the thirteen mushroom extracts on other cancer cell activity. Cells of A549, U87, Jurkat, HepG2, DU145, and Hela were inoculated in 24-well tissue culture plates at a density of the 8×10^4^ cells/well. Four hours after cell inoculation, extracts from the thirteen different mushrooms were added (400 µg/ml) and incubated for 48 hours. Cell survival was determined. n = 3, *p<0.05; **p<0.01.

We then tested the effects of these extracts on other types of cancer cell lines including the human adenocarcinomic cell line A549 (from ATCC), human leukemia cell line Jurkat (from ATCC), human prostate cancer cell line DU145 (from ATCC), human glioblastoma cell line U87 (from ATCC), human hepatocellular carcinoma cell line HepG2 (from ATCC), and human cervical cancer cell line HeLa (from ATCC). In general, *Amauroderma rude* exerted the most effective function in inducing cancer cell death (Fig. 3).

Amongst all medicinal mushrooms, *Ganoderma lucidum* and *Coriolus versicolor* are two of the most popular mushrooms used by cancer patients which are included in our study. *Ganoderma lucidum* is a popular medicinal mushroom with a reputation rivaling that of ginseng [24]. This mushroom has been widely used as a herb for the promotion of health and longevity in Asian countries. There have been reports on the biological activities including histamine release-inhibitory action [25], immunomodulatory activity [11], anti-tumour cytokine production [10], differentiation-inducing activity [26], and activity to induce cancer cell apoptosis and inhibition of cancer cell death [27]–[29]. *Ganoderma lucidum* has been widely used by patients with different types of cancers that are too advanced for surgery, chemotherapy, or radiotherapy. For those who have gone through one of these therapeutic treatments, *Ganoderma lucidum* is used as an alternative therapy, with an usual positive outcome.

The medicinal mushroom *Coriolus versicolor* is a macrofungi belonging to the *Basidiomycetes* class. It has been found that this mushroom has antimicrobial, antiviral and antitumor properties [30]. *Coriolus versicolor* is extensively used as an adjuvant in the treatment of different types of cancers. For over 30 years, Polysaccharide-Kureha (polysaccharide-K or PSK) and polysaccharopeptide (PSP) have been two major protein-bound polysaccharides extensively used as a chemoimmunotherapy agent in the treatment of cancer in Asia. Both molecules have documented anticancer activity *in vitro*, *in vivo* and in human clinical trials [31]–[34]. In both China and Japan, *Coriolus versicolor* extract is regarded as a valuable adjuvant for combination chemotherapy or radiotherapy in the treatment of various cancers.

Nevertheless, in our study, we found that the anti-cancer activity of both *Ganoderma lucidum* and *Coriolus versicolor* was significantly lower than that of *Amauroderma rude*. Since it was first described in 2007 (http://australianfungi.blogspot.ca/2007/04/7-amauroderma-rude.html), the name of *Amauroderma rude* can now be seen in scientific journal [35]. Currently, the fruiting bodies and spores of *Amauroderma rude* can be cultivated in wooden logs. It can also be obtained by fermentation to harvest mycelia. However, the function of *Amauroderma rude* is not known. Our study represents the first report for the function of *Amauroderma rude*. Compared with other well-known medicinal mushrooms, *Amauroderma rude* was found to possess the highest activity in inducing cell death of several invasive and metastatic breast cancer cell lines and other types of cancer cell lines. Understanding the physiology and biology of *Amauroderma rude* will allow insight as to whether or not this mushroom is suitable for human intake. With improved cultivation techniques, it has become possible to produce large quantities of *Amauroderma rude*. As more knowledge becomes available for this mushroom, it is anticipated that *Amauroderma rude* can soon be used as an adjuvant for health promotion, especially in cancer patients.

### Effect of *Amauroderma rude* on Inducing Breast Cancer Cell Death Compared with *Ganoderma lucidum*

**Figure 4:**
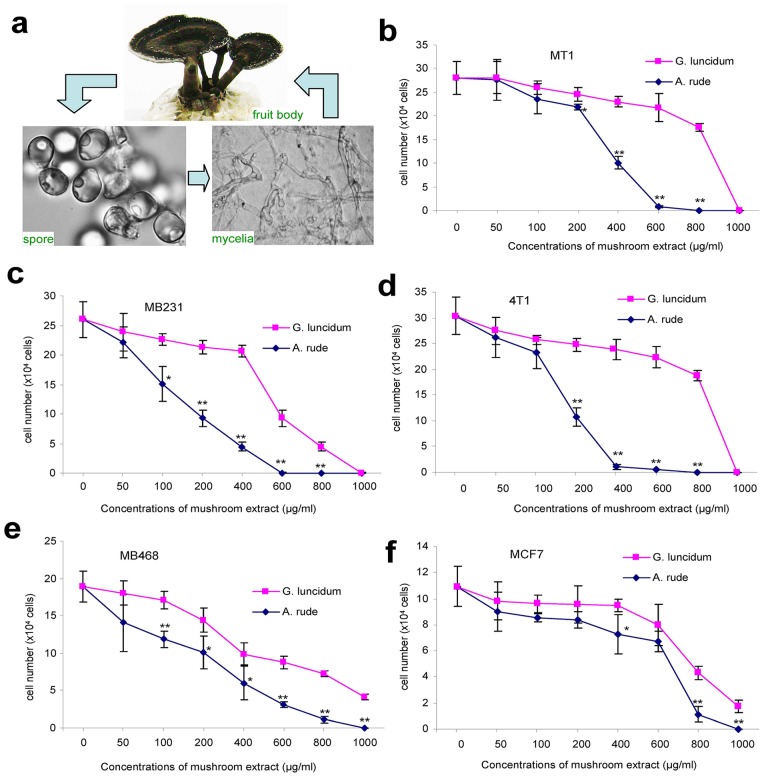
Effects of polysaccharide extracts from *Ganoderma lucidum* and *Amauroderma rude* on breast cancer cell death. The life cycle of *Amauroderma rude* contains spore, mycelia, and fruit body (a). Cells of MT-1 (b), MDA-MB231 (c), 4T1 (d), MDA-MB468 (e), and MCF-7 (f) were inoculated in 24-well tissue culture plates at a density of the 8×10^4^ cells/well. Four hours after cell inoculation, extracts from *Ganoderma lucidum* and *Amauroderma rude* were added to each well at different concentrations (0, 50, 100, 200, 400, 600, 800 and 1000 µg/ml) and incubated for 48 hours. Cell survival was determined by trypan blue staining and cell counting. n = 3, *p<0.05; **p<0.01.

The life cycle of *Amauroderma rude* is similar to that of *Ganoderma lucidum*, both of which belong to the same *Ganodermataceae* family. The typical life cycle of *Amauroderma rude* is composed of three living phases including spore, mycelia, and fruit body (Fig. 4a). As indicated above, *Ganoderma lucidum* is a traditional medicinal fungus used extensively in Chinese herbal medicine. Polysaccharides are the major source of its biological activity and the basis of its various therapeutic uses [2], [4]–[6]. We have previously reported that *Ganoderma lucidum* played an important role in inducing cancer cell death [15]. We thus compared in greater detail the effects of *Amauroderma rude* and *Ganoderma lucidum* on inducing death of breast cancer cells including the four invasive and metastatic breast cancer cell lines (MT-1, MDA-MB231, and 4T1), the less invasive breast cancer cell line (MDA-MB468), and the benign breast cell line (MCF7). We found that in the three invasive and metastatic breast cancer cell lines, there was a great difference of anti-cancer cell effect between *Amauroderma rude* and *Ganoderma lucidum* (Fig. 4b–d). No cancer cells could survive when they were treated with 600 µg/ml of *Amauroderma rude*. To reach the level of anti-cancer cell effect, we had to increase the concentration of *Ganoderma lucidum* to 1000 µg/ml, suggesting a 3-fold increase in the effect of *Amauroderma rude* on inducing cancer cell death as compared with that of *Ganoderma lucidum*. In the less invasive (Fig. 4e) and benign (Fig. 4f) breast cell lines, the difference in cell death induction between *Amauroderma rude* and *Ganoderma lucidum* was less evident.

### Effect of *Amauroderma rude* on Tumor Cell Survival, Apoptosis, and Colony Formation

**Figure 5:**
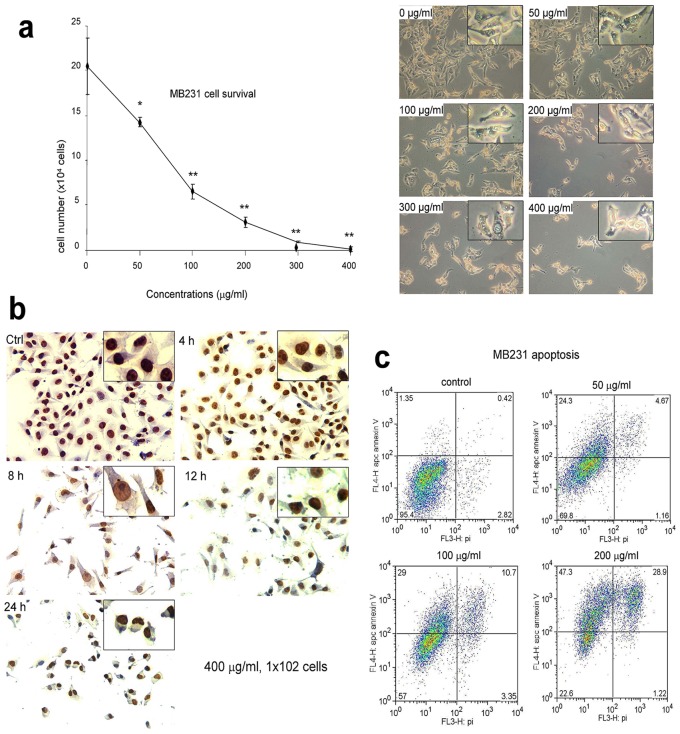
The effects of *Amauroderma rude* extracts on breast cancer cell survival and apoptosis. (a) The breast cancer cell line MDA-MB231 were cultured in normal medium treated with different concentrations of *Amauroderma rude* extract as indicated for 24 hours. Even at low concentrations, *Amauroderma rude* extract could effectively induce tumor cell death. Cell counting indicated a significant effect of *Amauroderma rude* at the concentration of 50 µg/ml (left). n = 3, **p<0.01. Typical photos of cell death are shown (right). (b) MDA-MB231 cells were cultured in normal medium treated with *Amauroderma rude* extract (400 µg/ml) for different periods of time. Extensive cell death was detected after eight hours of treatment. (c) MDA-MB231 cells were cultured in normal medium treated with different concentrations of *Amauroderma rude* extract as indicated for or 48 hours, followed by apoptosis assay. Treatment with *Amauroderma rude* induced apoptosis.

We then tested the effect of *Amauroderma rude* on tumor cell survival. The most invasive and metastatic human breast cancer cell line MAD-MB-231 cells were cultured and treated with different concentrations of *Amauroderma rude*. Even at low concentrations of 50 µg/ml it was found that *Amauroderma rude* exerted a significant effect on inducing breast cancer cell death (Fig. 5a). At higher concentrations (400 µg/ml), *Amauroderma rude* could induce cell death after 8 hours of treatment (Fig. 5b).

We examined whether or not *Amauroderma rude* could induce breast cancer cell apoptosis. MDA-MB231 cells were cultured in normal medium treated with different concentrations of *Amauroderma rude* extract. We found that at the low concentration of 50 µg/ml, *Amauroderma rude* exerted a great activity in inducing cell apoptosis as compared with the control (Fig. 5c, 29% vs. 1.8%).

**Figure 6:**
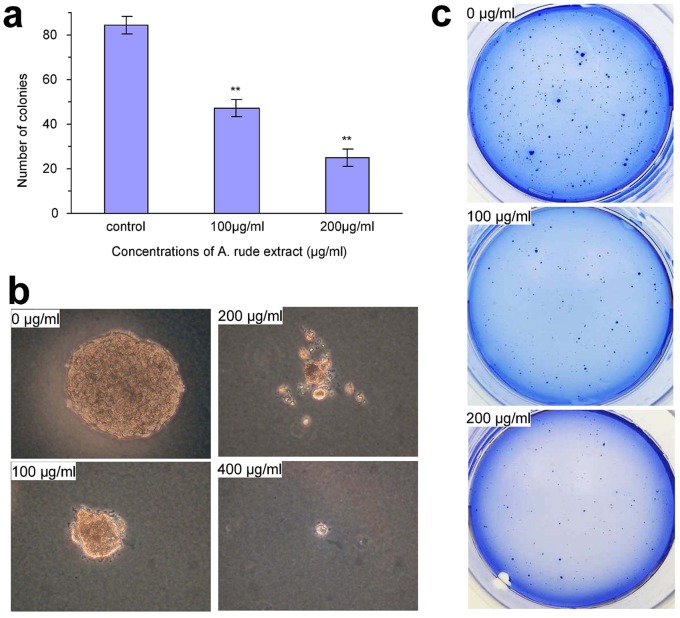
The effects of *Amauroderma rude* extract on colony formation. (a) MDA-MB231 cells were mixed with low-melting agarose at the density of 1000 cells/ml and cultured in normal medium. The cultures were treated with different concentrations of *Amauroderma rude* extract as indicated for 20 days. Colonies formed in the agarose gel were counted. Treatment with *Amauroderma rude* inhibited colony formation significantly (a). n = 3, **p<0.001. (b) Typical sizes of colonies. (c) Typical photos of culture plates containing colonies were stained blue.

Single cancer cells can form colonies in soft agarose gel. This is a hallmark of cancer metastasis and indicate the stem-like property of cancer cells. We tested whether or not *Amauroderma rude* could affect colony formation. MDA-MB231 cells were mixed with low-melting agarose and cultured in normal medium. The cultures were treated with different concentrations of *Amauroderma rude* extract for 20 days. Colonies formed in the agarose gel were quantified. Treatment with *Amauroderma rude* significantly inhibited colony formation (Fig. 6a). Careful examination of the colonies indicated that the colonies in the *Amauroderma rude*-treated plates were smaller than those in the control (Fig. 6b). To provide direct evidence of the inhibitory effect of *Amauroderma rude* on colony formation, we stained the plates with Coomassie blue (Fig. 6c).

### Effect of *Amauroderma rud*e on Tumor Growth *in vivo*

**Figure 7:**
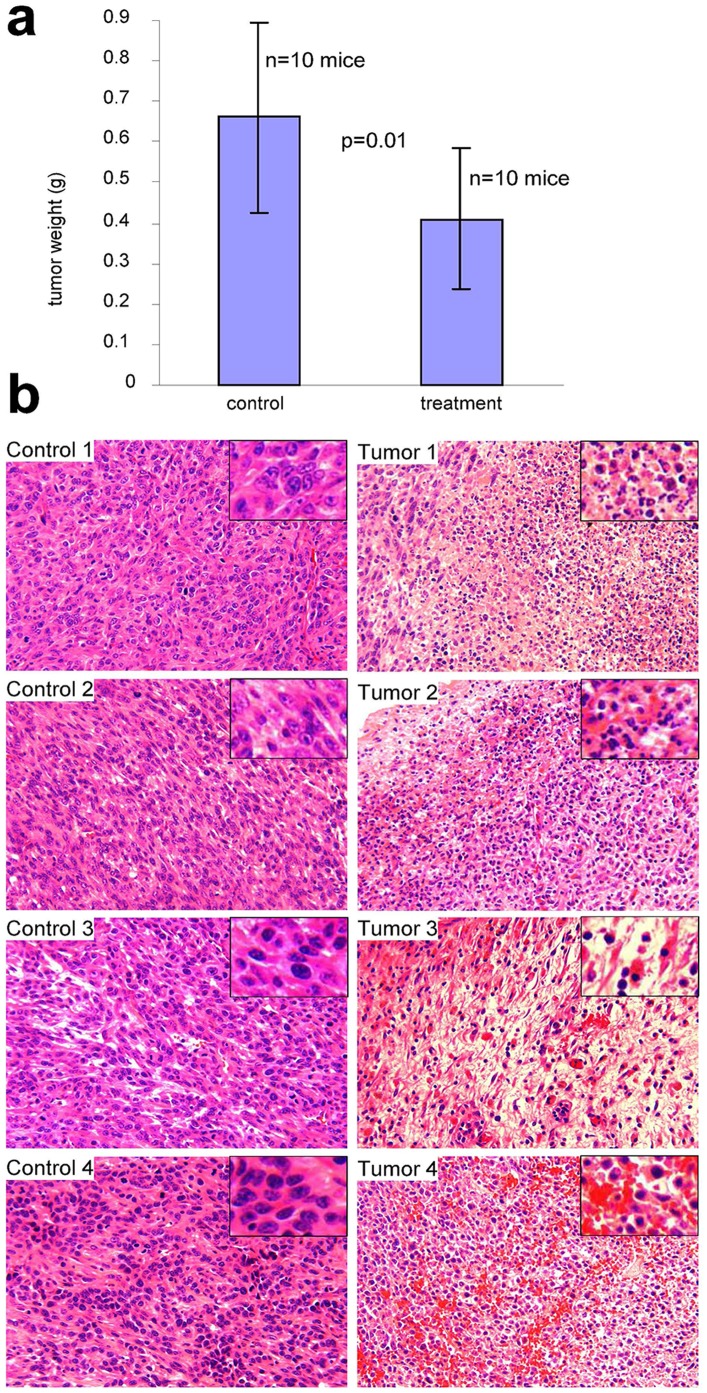
Tumorigenic activity of polysaccharide extracts from *Amauroderma rude*. (a) Balb/c regular mice were injected with 4T1 cells (2×10^5^ cells/mouse). Three days after the injection, mice were injected with *Amauroderma rude* (20 mg/site) locally or the buffer vehicle. This was repeated every other day. On days 15, mice were sacrificed and tumors were removed for further analysis. Treatment with *Amauroderma rude* reduced tumor sizes. (b) Tumor sections were subject to H&E staining and examined under a light microscope. Injection with *Amauroderma rude* induced extensive cell death and bleeding.

To validate the anti-cancer effect of *Amauroderma rude*, we developed a mouse model to test the extract of *Amauroderma rude*. Regular mice were injected with 4T1 cells, which can form tumors in strain Balb/c mice. Three days after the tumor cell injection, *Amauroderma rude* extract was injected locally in the location where tumor cells were injected. The extract was then injected every other day. On Day 15, the sizes of the tumors reached the limit set by the Animal Care Committee at Sunnybrook Research Institute and all mice were sacrificed. We found that treatment with *Amauroderma rude* significantly decreased the sizes of tumors (Fig. 7a). This *in vivo* result confirmed the anti-cancer effect of *Amauroderma rude*.

The tumors were sectioned and subject to H&E staining, followed by microscopic examination. The tumor sections in the *Amauroderma rude* treated group displayed extensive cell death, which was not the seen in the control tumors (Fig. 7b). We detected extensive nuclear condensation and fragmentation (Fig. 7b, Tumor 1). A great number of small fragmented nuclei were stained blue. Normally, cell death could always be seen in the center of a tumor. However, when the tumors were treated with *Amauroderma rude*, cell death could even be seen in the outer area of a tumor (Fig. 7b, Tumor 2). In some cases, the tumor cell density decreased greatly in the group treated with *Amauroderma rude* as compared with the control (Fig. 7b, Tumor 3). We also detected extensive bleeding in the tumors treated with *Amauroderma rude* (Fig. 7b, Tumor 4). The effect of *Amauroderma rude* on tumor growth appeared to be extensive, and this might explain the significant reduction of tumor sizes when the mice were injected with *Amauroderma rude* extract.

**Figure 8:**
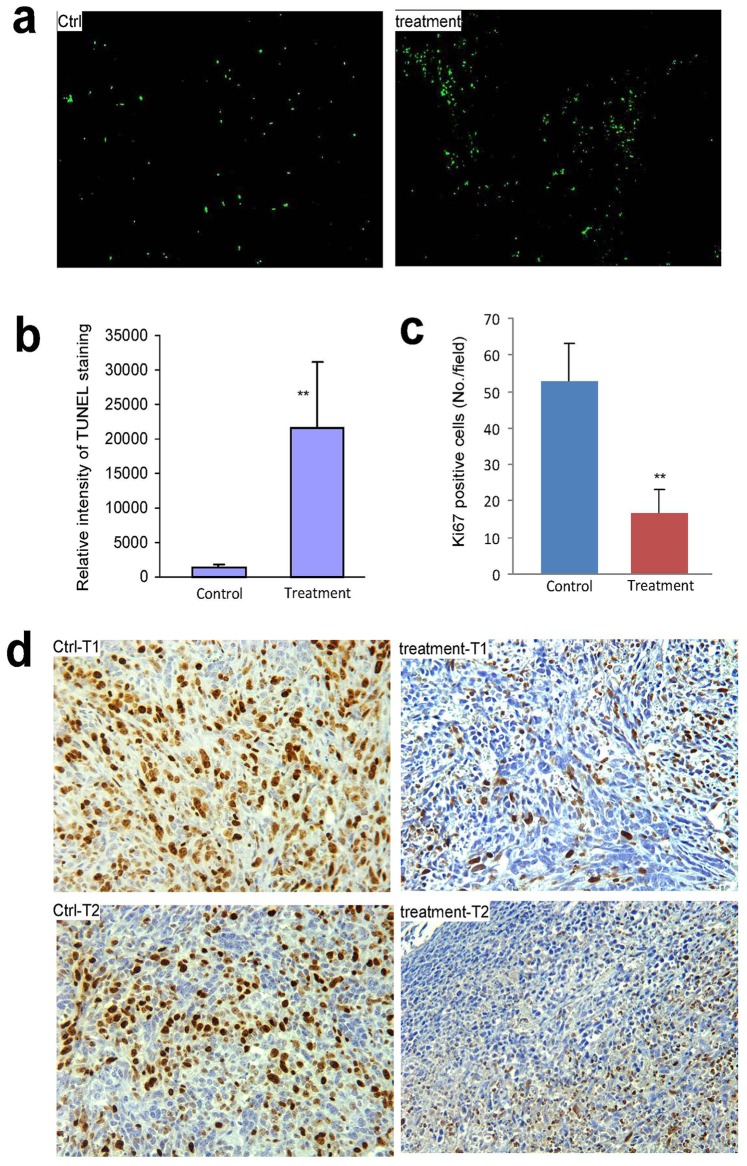
The effects of *Amauroderma rude* extract on tumor cell proliferation and apoptosis *in vivo*. (a) Tumor sections were subject to TUNEL staining and examined under a light microscope. Injection with *Amauroderma rude* induced cell death. (b) The TUNEL stained sections were scanned for quantification of apoptotic cells. Treatment with Amauroderma rude induced significantly more cell death than the control. **p<0.01. (c) Tumor sections were subject to Ki67 staining followed by examination and photograph under a light microscope to count the Ki67-positive cells. Injection with *Amauroderma rude* decreased Ki67 staining. (d) Typical photos of Ki67 staining are shown.

To examine the manner by which the tumor cells died, we performed TUNEL staining and found that the tumors treated with *Amauroderma rude* underwent apoptosis. A much greater density of TUNEL stained cells were detected in the treated tumors than the control tumors (Fig. 8a), suggesting apoptosis of the stained cells. The TUNEL stained sections were scanned with a software (ImageJ) for quantification. Treatment with *Amauroderma rude* significantly increased TUNEL staining (Fig. 8b). Since we detected extensive nuclear fragmentation of the treated tumors, there might be other types of cell death, i.e. cellular necrosis.

In addition, we also examined cell proliferation of the tumors using Ki67 staining. The stained Ki67 positive cells were counted. It was found that significantly fewer tumor cells underwent proliferation in the tumors treated with *Amauroderma rude* than that in the control tumors (Fig. 8c). There were weaker and fewer Ki67 positive cells in the *Amauroderma rude*–treated cells than that in control cells (Fig. 8d). These results suggest that *Amauroderma rude* exerted two layers of effect on the tumors *in vivo*: it could inhibit tumor cell proliferation and it could induce tumor cell death.

**Figure 9:**
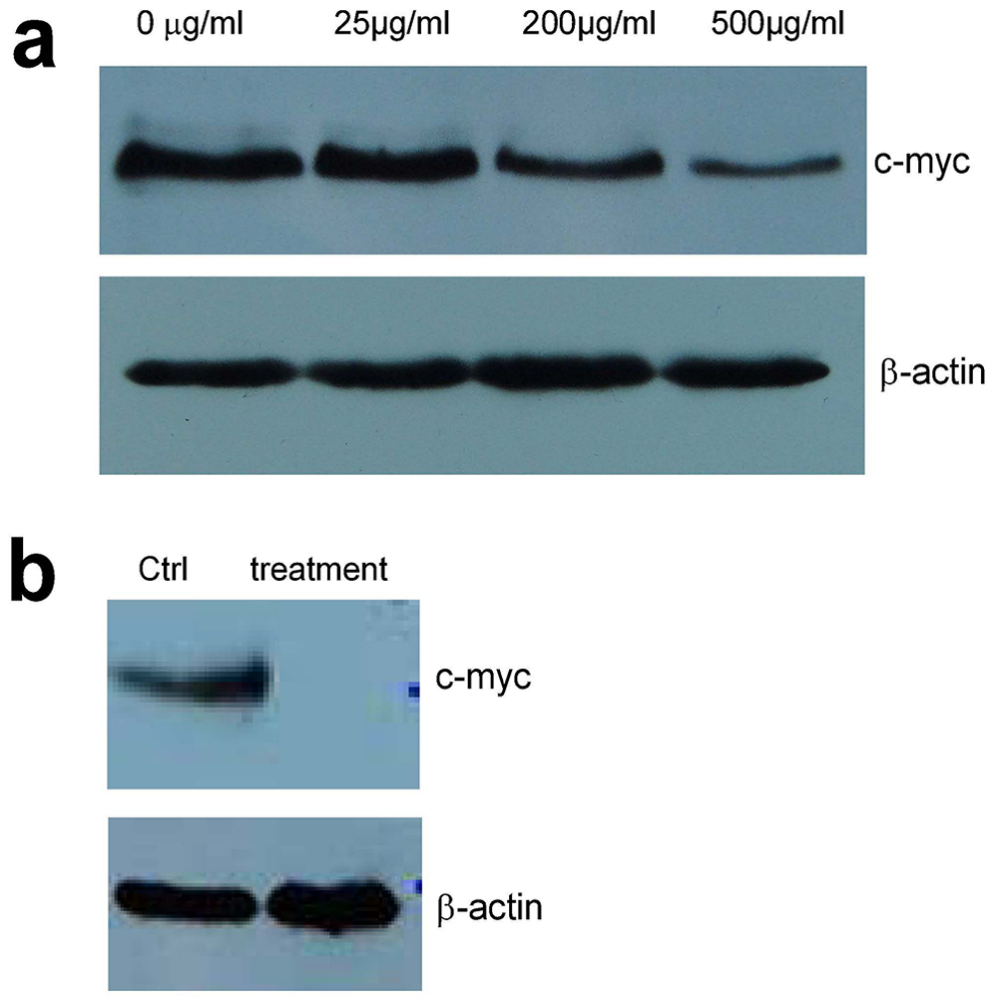
Suppression of c-myc expression by *Amauroderma rude*. (a) Cell lysate prepared from cells treated with or without *Amauroderma rude* was subject to Western blot analysis probed with anti-c-myc antibody. Expression of c-myc was suppressed by *Amauroderma rude* treatment. (b) Lysate prepared from tumors treated with or without *Amauroderma rude* was subject to Western blotting probing with anti-c-myc antibody. Expression of c-myc was suppressed by *Amauroderma rude* treatment.

To examine the signal pathway associated with these effects, we prepared cell lysate from cells treated with or without *Amauroderma rude* and analyzed protein levels in the lysate. We found that c-myc expression was suppressed in the cells treated with *Amauroderma rude* as compared with the control (Fig. 9a). Furthermore, we analyzed c-myc levels in the tumor lysate and found that treatment with *Amauroderma rude* inhibited c-myc expression (Fig. 9b). These results suggest that c-myc-associated pathway is responsible for *Amauroderma rude*-induced cancer cell death.

In summary, our study represents the first functional report for the medicinal mushroom *Amauroderma rude*. In all the thirteen mushrooms screened, *Amauroderma rude* possessed the highest activity in inducing cell death of a number of cancer cell lines. Compared with the two most well studied medicinal mushrooms, *Ganoderma lucidum* and *Coriolus versicolor*, *Amauroderma rude* displayed significantly higher activity in inducing cancer cell death. Notably, more than 2.5-folds the quantity of *Ganoderma lucidum* were needed to induce comparable levels of cell death of the most invasive and metastatic breast cancer cell lines MDA-MB-231, MT-1, and 4T1. Thus, *Amauroderma rude* shows promise as an alternative approach in treating cancer patients. Future studies will be undertaken to identify the bioactive ingredients of this mushroom possessing anti-cancer activity. Isolation and purification of the lead principle from this mushroom may set a milestone for the medical application of this mushroom. This will depend much on the structure, stability, and quantity of the lead compound. Chemical synthesis of the lead principle or the analogous will provide a means for drug development.

## Materials and Methods

### Cultivation of *Amauroderma Rude*

*Amauroderma rude* was cultivated on logs of wood of a mushroom farm in the Dabie Mountain in An-Hui Province. There are many mushroom farms in the area. One of them, owned by Mr. Jiang Qing-Wu, has been providing different types of mushrooms for us for many years. This has allowed us to obtain materials with high standard of quality control. In brief, the logs of wood had sizes of 5–10 cm diameter. Each log was cut to 14 cm in length. The logs were then partially dried to contain 65–75% of water. The logs were placed inside plastic bags, and the empty spaces were filled with culture media that contained sawdust (73%), wheat bran (25%), sugar (1%), and plaster (1%). The bags were closed and autoclaved. The mycelia of *Amauroderma rude* were inoculated into the media-containing logs of wood. Three weeks after inoculation, each bag was opened to have a small hole on one side allowing oxygen to get in. About two months later, the log-containing bags were ready for cultivation in a farm land, embedded beneath the soil. It took two more months to harvest the fruit bodies of *Amauroderma rude* when they were mature. The mushroom farm is located in the farm land area for cultivation of different types of mushrooms. It does not involve endangered or protected species. The owner of the farm (Mr. Jiang Qing-Wu) provided the research materials and gave permission to conduct the study on this site.

### Preparation of Mushrooms Extract

The extracts of different kinds of mushrooms were prepared from the fruit bodies using boiling water. It was performed at the Research and Development Centre of Guangdong Yue-Wei Edible Fungi Technology Co. Ltd. In brief, dried fruit bodies of mushrooms were ground into powders that could passed through a sieve with 60 pores per square inch. The powders (100 g each) were incubated with hot water (1∶10, w/v) in an incubator at 100°C for 2 h. After cooling down, the extracts were filtered and the filtrate was evaporated under vacuum to dryness using a rotary evaporator. The partially dried samples were then transferred to a drier that was maintained at 60°C till completely dried. The dried powders were used to calculate the extract yield. The assay was repeated once to confirm the results. The dried powders were stored at −20°C for later use.

### Cell Proliferation Assay

Human breast carcinoma cell lines (MB231, MCF-7, MT-1, and MB468), mouse breast cancer cell line 4T1, human adenocarcinomic cell line A549, human glioblastoma cell line U87, human hepatocellular carcinoma cell line HepG2, human prostate cancer cell line DU145, human cervical cancer cell line Hela, human leukemia cell line Jurkat, and human lung cells BEAS-2B, were used in this study. Cells were seeded on 24-well tissue culture plates at a density of 8×10^4^ cells/well in DMEM/RPMI 1640 containing 10% FBS. Four hours after cell inoculation, the extracts of 13 kinds of mushrooms were added to the cultures at a final concentration of 400 µg/ml. The medium used to dissolve polysaccharides served as a control. The cultures were maintained in a tissue culture incubator at 37°C containing 5% CO_2_ for 48 hours. Cells were harvested and mixed with trypan blue in a 1∶1 ratio for exclusion staining. Dead cells were stained as blue, while living cells were not stained by the dye due to the presence of the intact cell membrane. The number of living cells was counted. Each experiment was repeated three times for statistically analysis.

To compare the anti-cancer effect of water extract of both *Ganoderma lucidum* and *Amauroderma rude*, we performed proliferation assays using breast cancer cells. Cells were seeded on 24-well tissue culture plates at a density of 8×10^4^ cells/well in DMEM containing 10% FBS. Four hours after cell inoculation, the extracts of *Ganoderma lucidum* and *Amauroderma rude* were added to the cultures at different concentrations (50, 100, 200, 400, 600, 800 and 1000 µg/ml). The medium used to dissolve polysaccharides served as a control. The cells were maintained in a tissue culture incubator at 37°C containing 5% CO_2_ for 48 h. The cells were harvested and the viable cells were counted using trypan blue staining as above.

### Cell Survival and Apoptosis Assays

MDA-MB231 cells were seeded at the a density of 5×10^4^ cells/well in 24-well plates with 500 µl culture medium containing 10% FBS. Four hours after cell inoculation, cells were washed gently with PBS twice and cultured in serum-free medium. The water extract of *Amauroderma rude* was added to the cultures at different concentrations (50, 100, 200, 300 and 400 µg/ml). The medium used to dissolve polysaccharides served as a control. After 24 h treatment, the cells were harvested and the viable cells were counted using trypan blue staining as previously described [36].

An Annexin V-FITC apoptosis detection kit (Biovision Inc,Mountain View, CA, USA) was used to detect cell apoptosis. MDA-MB231 cells were seeded at the density of 5×10^5^ cells/well in 6-well plates with 2 mL culture medium containing 10% FBS. Four hours after cell inoculation, water extract of *Amauroderma rude* was added to the cultures at the concentrations of 0, 50, 100, and 200 µg/ml. After 48 h of incubation, the cells were collected and resuspended in 100 µl binding buffer, to which 5 µl Annexin V-FITC and 5 µl propidium iodide were added to each sample, and incubated in the dark for 20 minutes, After the incubation, an additional 400 µl binding buffer was added to each tube, and the cells were analyzed for Annexin V-FITC binding by flow cytometry (Ex = 488 nm; Em = 530 nm) using FITC signal detector (FL1) and for propidium staining by the phycoerythrin emission signal detector (FL2).

### Colony Formation *in vitro* and Tumor Formation *in vivo*

To analyze stemness-like activity of the cells, colony formation assay was performed. It was assessed using a method described previously [37]. In brief, 0.66% agarose gel was loaded on 6-well tissue culture plates. After solidification, MB231 cells (1×10^3^ cells/well) and water extract of *Amauroderma rude* (at the final concentrations of 0, 100 and 200 µg/ml) were mixed with 0.3% low-melting agarose in DMEM supplemented with 10% FBS, and plated on the top of 0.66% agarose. Colony formation and growth were monitored every other day. Twenty days after cell inoculation, colonies were examined under a light microscope.

For colony staining, the colony-containing agarose gel was prefixed with 80% methanol for 30 minutes, followed by staining with 0.25% Coomassie Blue (Bio-Rad) for 2–4 hours, until an uniform blue color was seen. The gel was destained with 5% methanol for 4–24 hours, followed by washing in 10% acetic acid until background is clear. Colonies were photographed under a light microscope.

Tumor formation in mice was performed using technique established in our laboratory [38], [39]. In brief, four-week-old mice purchased from Charles River were randomly divided into two groups. 4T1 cells (2×10^5^ cells/100 µl) were injected into six-week-old mice. The third day after cell implantation, water extract of *Amauroderma rude* was injected locally (20 mg/mouse). This was repeated every other day. Tumor growth was monitored twice each week. All of the mice were sacrificed by cervical dislocation and tumors were harvested on day 15. The animal experiments were performed according to the guideline approved by the Animal Care Committee at Sunnybrook Research Institute.

### H&E, Immunohistochemistry, and TUNEL Staining

Tumors formed in mice were freshly excised and fixed in 10% formalin. The fixed tumors were subsequently proceeded to be embedded in paraffin and sectioned. The sections were subject to H&E staining, immunohistochemistry and TUNEL staining by the methods described [40].

For TUNEL staining, an In Situ Cell Death Detection Kit (Roche) was used. The tumor sections were deparaffinized in xylene for 5 minutes twice, and then hydrated with 50%, 75% and 100% ethanol for three times. The sections were incubated in PBS-Tween 20 for 10 minutes and treated with Terminal deoxynucleotidyl Transferase (TdT) Reaction Buffer (Millipore) for 10 minutes. TdT Reaction Mixture were added to samples and kept for 2 hours at 36°C in humidified chamber, followed by rinsing sections in stop wash buffer for 10 minutes. Cell apoptosis were detected by incubating sections with Avidin-FITC in PBS for 30 minutes at room temperature. After Rinsing in PBS for 5 minutes, the slides were mounted with anti-fading mounting medium and subject to fluorescent microscope examination. At least four pictures were taken from different areas and green pixels were counted by using ImageJ (National Institute of Health).

The sections for immunostaining were performed by probing with antibodies against Ki67 (BD pharmingen, diluted at 1∶500) as described [13]. Before incubation with primary antibodies, peroxidase activity in the sections was inhibited with 3% H_2_O_2_ in TBS (10 mM Tris-Cl, pH 8.0, and 150 mM NaCl) at room temperature for 30 minutes. After washing with TBS containing 0.025% Triton X-100 for 3 times (3 minutes each), the sections were blocked with 10% goat serum for 1 h and then incubated with primary antibody in TBS containing 10% goat serum albumin overnight. The sections were then probed with biotinylated-secondary antibody at 1∶500 incubations, followed by avidin conjugated horseradish peroxidase (provided by the Vectastain ABC kit, Vector, PK-4000). The slides were subsequently stained with DAB followed by Mayer’s Hematoxylin for counter staining and slide mounting.

### Statistical Analysis

The results (mean values ± SD) of all the experiments were subject to statistical analysis by *t*-test. The levels of significance were set at p<0.05 and p<0.01.
